# Perceptions of Deaf education in the Deaf community

**DOI:** 10.1093/jdsade/enag036

**Published:** 2026-06-23

**Authors:** Gearóidín McEvoy

**Affiliations:** School of Law and Criminology, Maynooth University, Maynooth, Ireland

## Abstract

This article explores perceptions about education for the Deaf community within Deaf communities in Ireland, Finland, and the UK. Ireland, Finland, and the UK all provide legal recognition of sign languages in various forms. However, it is important to understand how these laws impact Deaf people, what their experiences with the law is, and how might the law be altered to improve the lives of people it purports to serve. Interview data from Deaf people across the three countries is presented here which explores these experiences. When asked about what they might improve in the law, an overwhelming number of responses related to the introduction of law to improve access to education. And so, this article has taken those responses and presented them for analysis. Research participants reported concerns about the quality of Deaf education, systemic problems in accessing education, acquisition of other language in Deaf people, the illusion of a choice of school options for parents of Deaf children and the dwindling number of Deaf schools. Participants suggested that meaningful access to bilingual education is needed for Deaf people.

According to the World Federation of the Deaf (WFD), 80% of Deaf people worldwide lack access to adequate education (Human Rights of the Deaf - WFD, 2024). However, even in those places where Deaf people do have access to education, often that education is lacking in quality and appropriateness for Deaf people (Human Rights of the Deaf - WFD, 2024). Deaf children, the majority of whom are born into hearing (and therefore, typically non-signing, non Deaf aware) families, lack access to sign language in the home and via school, which can result in language deprivation. As Gulati writes, language deprivation is an extreme rarity in the hearing world, but an “epidemic” among Deaf children (Gulati, 2019). Deaf people can also experience “sensorial asymmetry” whereby due to different access to education, they may have limited or contextual access to other languages (De Meulder et al. 2019). Meaningful, accessible, and appropriate education is a way to ensure language acquisition and prevent language deprivation in Deaf children. The types of education which are typically available to Deaf children include bilingual education, total communication, or oralism in either Deaf schools or mainstreaming education (World Federation of the Deaf, 2016).

This article looks at Deaf Communities in Ireland, Finland, and the UK. These three countries were selected for a number of reasons. First, Ireland and Finland share a similar overall population size and a similar Deaf population size. Ireland and the UK, however, share a similar overall culture and language, where English is a dominant language. Additionally, all three countries have a notable language minority with varying levels of official status (Irish in Ireland, Swedish in Finland, Welsh in Wales, Gaelic and Scots in Scotland, and attempts to give status to Irish and Ulster Scots in Northern Ireland). Finally, all three states have legislated for the recognition of signed languages in recent years, with Finland and Scotland legislating in 2015, Ireland legislating in 2017, and England and Wales legislating in 2022.

For this article, Deaf people were interviewed about their experiences with laws pertaining to sign language recognition. Overwhelmingly, participants mentioned that they wished for laws which recognize sign language to provide for better access to quality, bilingual education. As such, this article has presented that data and the context in which improved education access is sought in each jurisdiction. Education was discussed both in terms of the problems which exist for the Deaf community and by way of potential solutions to problems. Participants discussed the quality of education available, systemic problems for Deaf people accessing education, other language acquisition, and the dwindling number of bilingual schools for Deaf children. Suggestions were made as to how to improve systems, primarily through ensuring meaningful access to bilingual education. However, participants were also aware that even with the provision of excellent education on par with their hearing counterparts, barriers would still remain for Deaf people in society.

Prior to discussing the data findings, however, it is necessary to give some context for the state of education for Deaf people in Ireland, Finland, and the UK, and the relevant laws which relate to sign language.

## Educational contexts

Bilingual education involves teaching children through two languages one of which is sign language. Sign languages are the natural and native languages of Deaf communities, and when given the opportunities to learn, the most accessible languages for Deaf children (Barnum, 1984; Humphries et al., 2012; Ladd, 2003; Lane, 1999). Early exposure to sign languages has been shown to have benefits for the acquisition of other languages in Deaf children and language proficiency (Boudreault & Mayberry, 2006; Delcenserie et al., 2024; Mayberry et al., 2002) and impact their “cognitive, social and emotional development” (Courtin, 2000; Goodwin et al., 2022; M. L. Hall et al., 2017; Hauser et al., 2008; Langdon et al., 2023; Pontecorvo et al., 2023; Pyers et al., 2010; Pyers & Senghas, 2009; Schick et al., 2007; Woolfe et al., 2002; Zauche et al., 2016) While there persists some debate that sign language acquisition can somehow hinder the acquisition of speech and should therefore be avoided, systematic reviews of this literature found sign language acquisition had no impact on other language development and that studies claiming a negative impact were poor in quality (Fitzpatrick et al., 2016; Pontecorvo et al., 2023) There is also ample evidence to show that sign language deprivation in Deaf children can cause language deprivation (Gulati, 2019; M.L. Hall et al., 2017; W. C. Hall, 2017).

With historical roots dating back to 1880, oralism is also a method of education which can be used, which involves the prioritization of speaking and the prohibition of signing as an educational policy to the detriment of Deaf children (Glickman & Hall, 2018b; Ladd, 2003; Lane, 1999; Rose & Conama, 2018). Oralism has a long and painful history among Deaf communities worldwide.

Popularized in the 1970s, total communication is the ideology that Deaf children are provided with sign language, gesture, written, and spoken language to try and achieve maximum understanding (Jepsen, 2015). However, total communication has been criticized in the literature as actually impeding children’s ability to acquire language (Johnson et al., 1989; Ladd, 2003; Lynas, 2005).

Deaf schools or institutions may have been day schools, or residential boarding schools, or a mixture of both. Because Deaf children may be spread out across a country or region, and are not necessarily geographically proximate, it has often been necessary to establish boarding schools to cater to Deaf children who would otherwise not be able to commute home each day. Deaf boarding schools often became a site where Deaf culture and language could develop because it allowed Deaf people to exist in the same space and develop community (Lindberg, 2024; Murray et al., 2018). This can be irrespective of the educational context of the school. For example, evidence shows that despite a policy of oralism between 1892 and 1970s, *finlandssvenskt teckenspråk* (FSTS) was able to develop and be passed on from student to student in the residential boarding school in Porvoo, Finland (Lindberg, 2024).

Deaf schools are the typical location of education for Deaf children, although the prevalence of Deaf schools is on the decline in the three countries studied. Deaf schools were and are essential educational institutions where deaf children are educated. As will be shown in this paper, a Deaf school can employ any of the above educational modalities discussed.

Mainstreaming is now an increasingly popular form of education, where Deaf children are sent to otherwise hearing schools where spoken language is the medium of instruction and are given individual accommodations (Holt, 2003), using any number of the above educational modalities described above. While this may initially be viewed as beneficial and inclusive, it can be also seen as “the relocation of students for compliance with policy” rather than for the actual benefit of Deaf students (Komesaroff & McLean, 2006). In fact mainstreaming can mean Deaf children are increasingly isolated from their own communities and within hearing environments and is often objected to by the Deaf community itself (Baynton, 1996; Branson & Miller, 2010; Holt, 2003; Mathews, 2011, 2017).

There are various avenues for teacher training for teachers of the Deaf in each of the jurisdictions.

## Finland

As one of the first countries to recognize signed language (SL) in law, Finland has long been associated with Deaf Activism. The legal base and secretariat of the WFD is located in Helsinki and there has been two Finnish presidents of the WFD (Dr Liisa Kauppinen 1995–2003 and Dr Markku Jokinen, 2003–2011). In the Deaf community globally, Finland is often seen as “model country” for SL rights and SL-users (De Meulder, 2017).

The formal history of sign language usage in Finland can be traced to the establishment of a school for Deaf people in Porvoo in 1846 by Carl Oskar Malm (Kuurojen Museo (a), 2024; Takkinen et al., 2015). There are two distinct sign language communities in Finland: Finnish Sign Language (“*suomalainen viittomakieli*” SVK) and Finland Swedish Sign Language (“*finlandssvenskt teckenspråk”* FSTS).^1^ SVK is the more widely used of the two languages. FSTS is estimated to be in use by approximately 90 people in Finland, having suffered decline over the years (Katsui et al., 2021).

Oralism was the policy of education in Deaf schools in Finland from around 1900 until 1970 (Takkinen et al., 2015). In the 1970s “total communication spread to Finland,” taking over from oralism (Takkinen et al., 2015). However, as noted above, total communication has been criticized in literature as being an ineffective method of education (Johnson et al., 1989; Ladd, 2003; Lynas, 2005).

In 1995, sign language was recognized under the Constitution of Finland, without specific reference to the languages used. In the English translation, it states:

The rights of persons using sign language and of persons in need of interpretation or translation aid owing to disability shall be guaranteed by an Act.

That Act, the Sign Language Act, 2015 was the result of a campaign by the Finnish Association of the Deaf to introduce a law to better codify the rights of SL users when little the constitutional recognition had made little impact on the Deaf community (De Meulder, 2017). The Act recognizes both SVK and FSTS. De Meulder notes that the “Act is a framework law that does not contain any new rights or responsibilities for authorities” (2017). Section 4 of the Act, referred to as “Linguistic rights of sign language users.” Rather than actually providing any such rights, all three subsections of Section 4 merely refer to other acts.

In Finland, in order to teach a class of deaf children, a degree in special education is required (Working as a Teacher in Finland with a Foreign Qualification | Finnish National Agency for Education, 2025). This degree is specialized for teaching children with learning or other disabilities. A Deaf sign language user who trains to become a teacher for non-special education will, for example, not necessarily be qualified to teach Deaf students. As will be shown below, some participants to the research were unhappy with this structure. There have been calls to improve the standards of teaching in signed languages in Finland, ensuring teachers have a well-rounded understanding of sign language, Deaf culture, and community and pedagogy (De Weerdt et al., 2016).

Today, there are no official Deaf schools in Finland (Barish, 2024). A school network named Valteri, the National Centre for Learning and Consulting operates under the Finnish National Agency for Education hosts a unit in Jyväskylä called Onerva. Onerva has a dormitory for Deaf pupils, Deaf teachers, and focuses on bilingual Deaf education. Therein remote classes for Deaf children in SVK are provided for children across Finland (“Viittomakielen Verkkovälitteinen Opetus”, 2020). There are a number of mainstream schools which facilitate the teaching of Deaf students by Deaf teachers, but the availability has decreased. It seems that the Pitäjänmäki elementary school in Helsinki provides specialized classes for Deaf students using bilingual education (Barish, 2024). In her report on sign language pupil’s experiences of school in Finland, Selin-Grönlund notes multiple instances of students struggling in mainstream education where their participation in class is facilitated by an interpreter who renders the language of the teacher and other students into signed language for the Deaf student (Selin-Grönlund, 2021). Students who had access to Deaf education and students in mainstream education both experienced loneliness: the former feeling isolated from other Deaf children in their locality when outside school and the latter feeling isolated from other Deaf children in school.

## Ireland

Irish Sign Language (ISL) is the indigenous language of Deaf people in Ireland (Rose & Conama, 2018). Not all Deaf people in Ireland use ISL. For decades, ISL was discouraged or banned in institutions which educated and housed Deaf people, based on the belief that signing was detrimental to the ability to acquire spoken language (Rose & Conama, 2018). This policy, known as “oralism” prioritized spoken language acquisition in Deaf people, and discounted signed languages as actual languages (Gertz & Boudreault, 2016). Oralism in Ireland was introduced in the 1940s and 1950s, and continued until the 1990s (Conama, 2020). ISL was demonized in Ireland for many Deaf people. Rose and Conama (2018) show that even though an oralist method is no longer the educational policy for instructing Deaf children, it still remains an attitude present in Ireland. There is still a prevailing perception that spoken language—and in particular, English—is superior to ISL, and consequently, that ISL is inferior, rather than a complete language in itself.

Historically, two schools in Ireland were at the center of the Deaf community—St Joseph’s School for Boys, founded in 1856 and St Mary’s School for Girls, founded in 1846. In 2016, St Mary’s and St Joseph’s schools amalgamated into Holy Family School, which offers bilingual education of primary and post-primary Deaf children. The Midwest School for the Deaf, located in Limerick, was founded in 1979. The school teaches students “orally or through Irish Sign Language where appropriate” (Midwest School for the Deaf, 2024). Both Holy Family School and the Midwest School for the Deaf offer early intervention services, primary school, and secondary school. Holy Family School offers residential boarding for children.

A Bachelor of Education in ISL was launched in 2019 at the teacher-training facility at the Institute of Education at Dublin City University (Dublin City University, 2019). Prior to this, a post-primary grade in Irish was a requirement for all primary school teaching. While learning Irish is mandatory for students in primary and secondary education in Ireland, there are exemptions. Because of the mandatory oral and listening aspects to the state examinations in Irish, Deaf children are largely exempt from studying Irish. Without a grade in Irish, and without a command of Irish to teach children in primary school, most Deaf ISL users could not qualify as primary school teacher. No similar requirement for Irish competency exists for secondary school teachers, so in theory, Deaf people could train to become secondary school teachers alongside hearing students. However, it also ought to be noted that teacher training in Ireland requires an element of work placement, where student teachers are given teaching assignments and evaluated. Anecdotally, it can be difficult for Deaf student teachers to find work placements in hearing schools because there can be difficulty in accessing necessary accommodations in a hearing school. Finally, there is no requirement that teachers in Deaf schools where ISL is the mode of instruction be fluent in ISL. This has impacted the access to ISL and education for some Deaf children in these schools.

ISL was formally recognized in legislation in 2017 under the Irish Sign Language Act, 2017. Section 5 of the ISL Act refers to education supports for Deaf children. Section 5 legislates for schemes to provide ISL classes to family members of Deaf children and to provide ISL “support” to Deaf children in schools “pending the conclusion of the review of the Special Needs Assistant Scheme which the National Council for Special Education.”

## United Kingdom

In the UK, the dominant sign language is British Sign Language (BSL). Different dialects and variations of BSL are found throughout the UK. In Northern Ireland, BSL and ISL are used. In the UK, teachers of Deaf children are known as Qualified Teachers of Deaf Children and Young People or QToDs. (Quail, 2025) In order to become a QtoD, a teacher must already have what is known as “qualified teacher status”, according to the British Association of Teachers of the Deaf (BATOD) (Quail, 2025). This means that a QToD must first have a recognized teacher qualification, before acquiring additional training and qualifications to be recognized as a QToD. In applying to become a QToD, BATOD state that a candidate should have “a commitment to acquire basic sign language skills to Signature Level 1 or equivalent.” (Quail, 2025). Under 2023 Specifications for Mandatory Qualifications for QToDs, the UK Department of Education states that trainees must have a minimum of Level 1 BSL (beginner level), with plans to pursue Level 2 within 3 years. If working with a predominant BSL user, at least Level 3 BSL is required. (Mandatory Qualifications: For Specialist Teachers of Children and Young People with Hearing Impairments, 2023).

According to the National Deaf Children’s Society, today there are 22 schools for Deaf children in the UK. As the UK has a much larger population, consisting of four nations, and has considerably more schools than Ireland and Finland, Table 1 depicts all of the schools and their educational contexts.

**Table 1:** Breakdown of educational facilities for Deaf children in the UK

School name	Location	School type	Education method
Elmfield School for the Deaf Early Years Primary and Secondary	Bristol	Nursery, primary, and secondary	Bilingual—BSL and English
The Deaf Academy	Devon	Primary, secondary, and further education	Bilingual—BSL and English
Mary Hare School	Berkshire	Primary, secondary, and further education	Oral—English
Hamilton Lodge School and College	East Sussex	Primary, secondary, and further education	BSL, SSE, English (total communication)
Frank Barnes School for Deaf Children	London	Nursery and primary	Bilingual—BSL and English
Blanche Nevile School for Deaf Children Primary and Secondary	London	Nursery, primary, and Secondary	Bilingual—BSL and English
Oak Lodge School^a^	London	Secondary	BSL, SSE, English (total communication)
Heathlands School	Hertforshire	Nursery, primary, and secondary	Total communication
Knightsfield School	Hertforshire	Primary, secondary, and further education	Oral—English
Royal School for the Deaf Derby	Derby	Nursery, primary, and secondary	Bilingual—BSL and English
Longwill School for the Deaf	Birmingham	Nursery and primary	Bilingual—BSL and English
Braidwood Trust School for the Deaf	Birmingham	Secondary	Total communication
St John’s Catholic School for the Deaf	West Yorkshire	Nursery, primary, secondary, and further education	Oral—English
Doncaster School for the Deaf and Communication Specialist College^a^	Doncaster	Nursery, primary, secondary, further education, and college	Total communication
Royal Cross Primary School	Preston	Primary	Total communication
Thomasson Memorial School	Lancashire	Nursery, primary, and secondary	Oral, SSE, BSL
Royal School and College Manchester^a^	Manchester	Primary, secondary, further education, and college	BSL in addition to specialist teaching
Hedleys Northern Counties School^a^	Newcastle upon Tyne	Nursery, primary, secondary, and further education	Total communication
Aberdeen School for the Deaf	Aberdeen	Nursery, primary, and secondary	BSL, SSE, English (total communication)
Windsor Park School for the Deaf^a^	Falkirk	Primary and secondary	Total communication
Hamilton School for the Deaf	Hamilton	Nursery and primary	Total communication
Jordanstown School^a^	Antrim	Primary, secondary, and further education	Total communication

^a^Denotes schools which are specialized to cater to children with additional disabilities

The schools catered for a range of ages, from nursery, right up to further education and college, with most schools catering to more than one age range. Five schools are specifically for children with disabilities, including Deaf children with additional needs. Notably, there are no schools for the Deaf in Wales. There are three schools for the Deaf in Scotland. There is only one school for the Deaf in Northern Ireland, and this school is a school for Deaf, Deafblind and blind (non-Deaf) children.

In terms of types of education, it is clear from Table 1 that the majority of schools operate using total communication. As noted above, total communication favors use of sign, speech, and gesture to facilitate language acquisition. However, Moores notes that “total communication” can also be used to describe instances where sigh supported English (SSE) is used to support spoken English acquisition in the absence of any sign language (2010). As such, in Table 1, schools which describe their education methods as being on a spectrum of BSL, SSE and English have been understood as using total communication. It is unclear to what extent a descriptor of “total communication” actually supports BSL acquisition in each school, and that data is beyond the scope of this work. Nevertheless, 54.5% of schools (12 of 22) adopt a total communication approach or similar.

Six schools (27.3%) described themselves as being bilingual in BSL and English. Bilingual education would ensure children have access to BSL as a language of instruction, rather than the imposition of English grammar on BSL signs, as described by Ladd above. Two of the bilingual schools are in London, and one each is located in Devon, Bristol, Birmingham, and Derby. One school, (4.5%) a school for children with additional disabilities, described its teaching method as using BSL for children who respond best to sign language, in addition to specialist teaching methods (The Seashell Trust, 2024). Three schools (13.6%) attested to using an oral method of communication. Among those schools, there was use of somewhat alarming and outdated language to describe Deaf children. One school notes in its “About” section:

St John’s is an oral school where pupils are taught by specialist teachers of hearing impaired children. There is great emphasis on supporting the development of pupils’ spoken language as well as reading and writing. (“St John’s,” 2024)

The language of “hearing impairment” is typically not used when describing Deaf people and represents harmful stereotypes, as described by the UK-based Deaf health charity, Sign Health “In some settings you might see the term ‘hearing-impaired’ but many people find being labelled ‘impaired’ offensive and inaccurate, so we don’t use that term.” (“What Is the Difference between Deaf and Deaf?”, 2025). The school website goes on to state that “[m]ost pupils arrive with linguistic levels significantly below their hearing peers.” Certainly, many Deaf children do experience linguistic levels which are lower than their hearing peers. Research shows, however, that this is largely due to language deprivation syndrome, (Glickman & Hall, 2018a) particularly when oralism is preferred over signed language acquisition.

There are two pieces of legislation which recognize BSL in the UK: The British Sign Language (Scotland) Act 2015 (BSL Scotland Act) and the British Sign Language Act 2022 (BSL Act). The BSL Act makes no reference to education, and in fact section 1.2 prevents the Act from having any impact on other pieces of legislation. Therefore, although the BSL Act recognizes BSL as the language of England, Scotland, and Wales in section 1.1, this recognition is barred from having any real impact. Similarly, the BSL Scotland Act does not reference education or provide any right for Deaf children to access sign language education. Rather the function of the Act is to require authorities to produce “National Plans” for the promotion of BSL. As of the time of writing, there is no law recognizing BSL and ISL in Northern Ireland.

From this point, we can see what the current status of bilingual education for Deaf children in Finland, Ireland, and the UK. Following a discussion of the methodology used to collect interview data, I will explore RQ1 and present how people in Finland, Ireland, and the UK perceive the status of education for Deaf people.

## Methodology

Access to language is a crucial issue of concern, therefore for Deaf people and for Deaf children. As such, this article asks three research questions:

RQ1: How do Deaf people in Finland, Ireland, and the UK perceive the status of education for Deaf people?

RQ2: What changes or improvements to the provision of education for Deaf people in Finland, Ireland, and the UK are suggested by Deaf people in those countries?

This research was informed by qualitative data collected during semi structured interviews with Deaf people from Ireland, Finland, and the UK, wherein they were asked about their experiences of legal recognition, and their hopes for change. Deaf participants were selected, as the laws pertaining to protect sign languages inevitable impact the Deaf community. Sign languages are the natural and native languages of Deaf people and Deaf rights are often inherently linked to the introduction of laws to protect and promote signed languages (Human Rights of the Deaf - WFD, 2024).

The research design underpinning this work was hermeneutic phenomenology, which can be understood as being:

concerned with the life world or human experience as it is lived. The focus is toward illuminating details and seemingly trivial aspects within experience that may be taken for granted in our lives, with a goal of creating meaning and achieving a sense of understanding. (Dibley et al., 2020)

Hermeneutic phenomenology^2^ allowed this work to be led by the lived experiences of the Deaf participants and to find meaning in their experiences. Hermeneutic phenomenology is a reflective process which encourages the researcher to confront their own identity, lived experiences and biases and then to use the understanding of those experiences as a tool in the research itself (Conama, 2022). I am hearing, and have English as a first language and therefore an outsider to the Deaf community (Dibley et al., 2020). However, I have also been researching the Deaf community for close to a decade and have spent a large portion of that time learning ISL, have many friends and colleagues who are Deaf and would consider myself Deaf Aware. This connection could be considered by some as being an “insider” to a community (Davis, 2015). Ultimately using hermeneutic phenomenology to analyze my own lived experiences, I consider myself outside the Deaf community, but with knowledge of the community and a commitment to trying to be an ally to the community. This position allowed me access to the community and allowed me to demonstrate a shared understanding with participants and empathy in the data analysis. Hermeneutic phenomenology is a continuous process throughout the research project whereby I was constantly reflecting on my own position and experiences and how those experiences are in flux with those displayed in interviews. Importantly, hermeneutic phenomenology used by a different person, interviewing the same people, at the same time, asking the same questions would possibly yield different results. That is not to say that these results are methodologically unsound, but rather they are subjective, and that subjectivity is valid and acknowledged as a benefit in this work. It is because of my own lived experiences (as opposed to in spite of them) that I as a researcher am able to connect with people, that I am able to understand Deaf lived experiences and that I am able to produce these findings.

The goal of this work was to investigate lived experiences of Deaf people under laws which recognize sign language in law. Only by asking Deaf people—who are necessarily most concerned with such laws—can we understand how the laws work and what ought to be changed in the future. As such, hermeneutic phenomenology gave the space and opportunity to seek out and examine those vital lived experiences and center them in the research. It is through an examination of the lived experiences in this way that this paper can present the specific needs of and experiences from Deaf people in relation to education. The collected data showed an overwhelming concern among participants in all three countries regarding education, and as such, this paper is centering those concerns.

Flowing from this goal of lived experience research and the chosen design, semi-structured interviews were chosen as the method because they represent a compromise between interviewer led and interviewee led communication and allow for a more conversation-type interview than structured interviews (King & Wincup, 2008). This would allow the author to direct the conversation through open-ended questions but ensure the interviewee could have the freedom to present their own narrative on their lived experiences (Dibley et al., 2020). To access lived experiences, it was necessary to allow participants the space to control what they wanted to discuss and focus on the issues that were most important to them.

Interviews were conducted between May and November 2022.

### Participants

Interviewees were recruited through a series of video calls for participation in ISL, BSL, SVK, and FSTS posted on social media and online. Deaf adults living in Ireland, the UK, and Finland were sought without limitation as to age, background, status, etc. Those who responded to the calls were interviewed after informed consent was secured. See Table 2 for a breakdown of the names, locations, and language used by participants.

**Table 2:** Research participants

Finland		Ireland		United Kingdom	
Pseudonym	Language	Pseudonym	Language	Pseudonym	Language
Aino	SVK	Gráinne	English	Oliver	English
Benita	English	Frances	ISL	Richard	BSL
Kerttu	SVK	Matthew	ISL	Andy	BSL
Mikko	SVK	Fiona	English	Jack	BSL
Otto	SVK	Martin	ISL	Meghan	BSL
Juha	SVK	Cillian	ISL	Charles	BSL
Tiina	SVK	Cathal	ISL	Paul	BSL
		Dara	ISL	Alan	BSL
		Anna	ISL	Vivienne	BSL
				Sarah	BSL
				Tim	BSL
				Peter	BSL

Participants were given pseudonyms for their participation in this work which generally reflect their presented gender and their country of origin, but it should be noted that personal gender identity, age, ethnic origin, or race was not interrogated by in interviews due to the nature of the interview style. For context, the group consisted of participants who presented racially as white, who appeared to range in ages from early 20s up to 60s, and ranged across gender. Certainly, some participants referred to their gender, age, race, etc., but I did not explicitly seek that information and therefore cannot provide that data on all participants beyond speculation. Twenty-nine interviews were conducted: Ten with Irish participants, twelve with participants from across the UK and seven with Finnish participants. One Irish participant was excluded for concerns about their consent to participate. As such, the dataset stands at 28 participant interviews. All participants were Deaf. Participants included Deaf academics, health workers, media professionals, educators, legal professionals, community workers, activists, policy workers, and employees of advocacy groups, and charities for Deaf people. As such, all participants were considered as having expertise in understanding the needs of their respective Deaf communities.

As noted above, I am hearing with English as a first language. I have an intermediate command of ISL and no command of any other signed language. However, in the purposes of these interviews, it was thought to be better to avail of interpreters, even when interviews were to be conducted in ISL, to assure maximum understanding for all parties. Four participants (two from Ireland, one from UK, and one from Finland) requested to be interviewed in English, without the use of an interpreter. Only one participant was not a sign language user but was learning to sign. They were included in the dataset, however, as they represented the experience of people who are Deaf but for various societal and historical factors, have been denied access to signed language. Sign language is the language of Deaf people, irrespective of whether they have learned their language.

### Data capture

Twenty-four participants used interpreters, and all interviewees were given their choice of interpreter (although some interviewees had no preference). All interpreters were certified and/or registered on their relevant country’s registry of interpreters, ensuring quality and trust in the interpretation. Interpreters were contacted directly, as contact details were always available from the relevant country’s register. Two interviews were conducted and recorded in person. The remaining interviews were conducted and recorded with the participant’s permission over Zoom. Zoom was used an accessible option to interview participants all across the three states chosen. When considering the often sparse availability and distribution of interpreters across each state, having Zoom as an option allowed for more flexibility in scheduling, as well as lowering costs associated with international travel in order to conduct interviews. Interviews were semi-structured and carried out using English and ISL, BSL, or SVK. Interpretation was rendered then into the target signed language and vice versa. Interviewees discussed their experiences with the sign language acts in their jurisdiction. In the case of Northern Irish participants, where there is yet no law on sign language, participants discussed the proposed law, their perspectives on the law in other jurisdictions and what they would like to see from the law.

At the end of each interview, participants were all asked for their suggestions of how to improve the law if they deemed it necessary. When asked, an overwhelming number of responses related to the introduction of law to improve access to education. And so, this article has taken those responses and presented them for analysis. Education was suggested by participants as a way to improve the experiences of the Deaf community under laws recognizing signed language and protecting the Deaf community. Participants often wished that laws provided funding for education and meaningful access to signed language.

### Data analysis

Interviews were transcribed and analyzed in Nvivo 13 for Mac. Nvivo is an extremely useful software for analyzing qualitative interview data and can help in the visualization of trends across data (Bazeley & Jackson, 2013). Member checking was not possible in the research, due to the multilingual, multimodal method of data collection. Interpreters interpreted all signed languages into English, and the transcripts were then rendered in English from these recordings. It could not be then guaranteed that all participants were sufficiently competent in written English to check the transcripts. Rather validity of the transcript was assured through the use of trained, registered interpreters, most often chosen by the participant as a trusted interpreter. In analysis, interviews were first read in depth and then read a second time to identify recurring themes which arose. Themes were classified with “nodes.” For example, there was a node for “education” for each time an interviewee mentioned education. There was also a node called “ideal changes” where participants discussed what they would change in the law. Underneath this node were subcategories, called “child nodes” which included “education” for when interviewees stated the changes they wished to see related to the provision of education. This type of coding was done through repeated analysis of the transcripts and consultation with other members of the research team (Bazeley & Jackson, 2013; Saldaña, 2013). Upon analysis of the interview sections coded to education, the data was sorted into themes and five main themes arose, which have been listed here in the following section. Pseudonyms have been given to all participants in the data to help protect their identity and maintain their anonymity.

## Results

From the data, one of the major issues of concern for participants across all three jurisdictions was education. Twenty-three of the participants refenced education although not every person is quoted in this article as not every quote fell under the major themes from the data discussed in this article. Within that, participants referred to problems with the quality of education, the acquisition of other languages, systemic problems, the illusion of choice, the dwindling number of Deaf Schools, and meaningful access to sign language education. However, participants also noted that even with better access to education for Deaf people, barriers to equality would still remain for Deaf people and these barriers ought to be considered.

### Quality of education in Deaf schools

Many participants were concerned that the provision of Deaf schools, or bilingual education does not necessarily ensure quality teaching. This was consistent across Ireland, the UK, and Finland. First, the Finnish participants noted:

Tiina: In Helsinki, [there is] one school with, I think there's five or six classes and they use sign language. But the quality of education is questionable. And I understand why, because you know, in year one, you are presented with these children, and the language levels are very varied. Some have come from Deaf families, which means that you've got fluent signers. Whereas some are from hearing families and maybe they've got no language whatsoever. They might be seven, and they're starting school… And maybe they're starting with no signed or spoken language whatsoever. So how do you teach that mixed group? You're trying to teach them the same information and that's really difficult. And of course, that will affect the quality of the education that you can provide. So that is something that I am very concerned about.

Tiina acknowledged that the variation in language capabilities of children in schools can be a barrier to quality teaching. These same multifaceted problems which can interfere with teaching have been found in literature elsewhere. Deaf children may be coming from a variety of different linguistic backgrounds or they may be enrolling in Deaf schools because they have other language delays (Dammeyer & Marschark, 2016; Shaver et al., 2014).

Otto was concerned that teachers of Deaf lack appropriate training:

Otto: [In Finland] many of them [teachers] may not be qualified to be a teacher of the Deaf, to be able to make sure that Deaf children are able to achieve their potential, to be able to achieve the necessary qualifications. So, it’s not always the fault of the Deaf child. It could be fault of the system. And training teachers to be able to teach.

Aino shared this concern, noting that there is no assessment for sign language capabilities for teachers of Deaf children.

Aino: In Finland as well, we don’t have any regulations or even – we don’t even have tools to assess their teachers’ sign language skills.

Mikko noted that there was concern among the Deaf community about the quality of education in Deaf school.

Mikko: [I know Finnish] families of Deaf parents…who don’t want to send their children to the Deaf school because of the level of education. According to them it is not great.

Attainment in Deaf schools has been shown to be lower, but again, there are a variety of factors which affect this, including teacher quality and language (Dammeyer & Marschark, 2016; Shaver et al., 2014). In the UK, one participant shared the concern that teachers of the Deaf were not competent in BSL and therefore would struggle to educate Deaf children through BSL.

Andy: And, most of them [teachers in Deaf schools in the UK] can’t sign well - the theory is that most of them can sign, but actually it's not always the case, and when I tell people that they're just really shocked by what. ‘What do you mean they can't they can't sign?' When really, they can't. I mean I have Deaf children myself. And it's been a real, I’ve had a real hard time communicating with their teachers, because they can't sign, so I have to really slow my signing down and make it really basic in order for them to understand me and they're actually teaching my children.

In acknowledging the importance of community for Deaf children in Deaf schools, Vivienne noted that this can mean sacrificing quality education:

Vivienne: I think it's good for children to have a group, so they don't feel that they are isolated, you know, that they are, that they can mix and socialize. But I am concerned about the quality of education. Because I am aware that a lot of people sort of, teenagers, they go to Deaf schools [in the UK] and the education system is quite poor.

In Ireland, one participant who was a sign language teacher expressed concern for the lack of a register for sign language teachers.

Martin: And what about [Irish] sign language teachers [they are] not registered anywhere you don't get that kind of status.

Martin here is comparing to the existence of a register for ISL interpreters under the ISL Act, the Register of Irish Sign Language Interpreters (RISLI). All ISL interpreters working in Ireland must be registered on RISLI. There is a minimum education requirement, quality monitoring, and a complaints mechanism under the terms of RISLI. However, there is no standard for ISL teachers. In theory, anyone can advertise themselves as an ISL teacher. Martin was concerned that the quality of ISL teaching because of the lack of a monitoring body.

Cillian referenced his own experience in third level education:

Cillian: I remember when I was [in third level education]. And I was applying for access to you know reasonable accommodation, all of those kind of things to get interpretation, but it was a little bit of contention there over it. I had some ISL provision, but also about note taking I had to pick one or the other, so I think you know if you have to pick either or it's difficult. I was sharing the course with another Deaf student so the other student took the note taker, and I took the interpreter that's how we so we could have shared the provision of resources.

In this instance, Cillian felt himself lucky to be able to share resources with another Deaf student in order so that they both might have maximum access. The system in place allowed for either an interpreter or a notetaker, without an understanding of how both resources would ensure better quality of education for a Deaf student.

The quality in education for Deaf students often went hand in hand with concerns about systemic problems which impede access to education.

### Systemic problems

From the data, participants felt that systems are in place which actively prevent Deaf people from accessing education or becoming teachers themselves and providing education to Deaf students. Vivienne noted that in the UK, due to the quality of education and barriers to language acquisition, Deaf students would struggle to overcome university entry requirements:

Vivienne: Now if people fail their GCSE English, you can't get funding to go to university. So that would mean Deaf students can't go to university because most of them won't pass their first time [because of poor access to English language]. I know a lot of Deaf people, it's shocking, really. But the school doesn't offer them an English GCSE.^3^

Again, referring to third level education in Ireland, Cillian noted how there is a need to repeatedly fight for access and provision, even when those provisions are an entitlement:

Cillian: [In order to access higher education in Ireland] you know you have to kind of persevere with each disability officer and explain over and over again to them as to what provision, so I think there should be some kind of guidelines about what is equitable provision and what's an acceptable standard.

Despite working with disability offices, Cillian alluded to a lack of knowledge about Deaf students therein.

Peter referred to the perception of Deaf children as disabled as a barrier to appropriate education in the UK:

Peter: I think a big problem with the education [in the UK] is that the legal side of things where it says that Deaf children—a Deaf child is special or additional needs, that's what they're labelled as, so they need additional needs, additional support learning. Some children absolutely do, of course they do if they need additional support with their learning. But if you look at a children who is just Deaf, that's it, then they need BSL. You need sign language.

Certainly, many of the schools listed in Table 1 are also schools for children with additional needs or disabilities. But Peter worried that conflating deafness alone with disability education hindered the progress of Deaf children. This is can be seen in the literature, where positioning Deaf children as disabled can medicalize their experience and can ignore their linguistic needs in favor of a disability perspective (Mathews, 2011).

Many participants were concerned about the criteria for becoming a teacher, and the barriers that Deaf people need to overcome in order to qualify as a teacher. In Finland, Aino complained about how a teaching qualification was insufficient, and a degree in teaching children with disabilities was required:

Aino: [I] studied myself to be a normal [sic] teacher, I couldn’t go and teach Deaf children because I don’t have the education of the…teachers of special needs kids need to have.

Aino was denied the opportunity to teach Deaf children, despite being a qualified teacher and despite having fluent SVK, because of the systemic position that Deaf children are disabled and require specialist education as such. This may also feed into some of the concerns about the quality of teaching in Deaf schools as presented in the previous section. Teachers may be systemically more equipped to teach children with disabilities generally and not prepared to teach Deaf children using a signed language (Shaver et al., 2014).

Similarly in the UK, Vivienne complained that in order to qualify as a teacher, students must complete a traineeship at a hearing school, rather than at a Deaf school, irrespective of the intentions of the student:

Vivienne: Because one of the criteria [for completing training as a teacher in the UK] is that you have to work in a normal [sic] school. So that means you go and work with hearing children. Okay. But I don't, you know, I only want to work with Deaf children. That's my aim. That's what I want to do. So that's—and then on top of the funding for a teacher of the Deaf, if that's what you wanted to become, you have to pay for an additional course. Then on top of your teacher training, you have to have a job in a Deaf school.

She noted that additional costs are also incurred if students do wish to become a teacher of the Deaf. The cost of formal qualifications was also a concern for Paul. Here Paul is discussing the proposed introduction of BSL as a GCSE (state examination). Paul was concerned that while the intention was positive, there are insufficient Deaf teachers to meet the demand:

Paul: And if the GCSE comes through, if, then that means you’ll need people qualified to be a qualified teacher and most of the people aren’t. Most Deaf people who teach BSL have got like a City & Guilds and that’s not good enough to teach in school. It’s not enough. Doesn’t match the criteria. They wouldn’t be allowed to teach children in a school environment. And then they’d have to be told they need to go on a course and of course you can’t afford the money. Who’s going to pay for it? [indicates that likely it will be hearing people who end up having the teaching qualifications, Deaf people will be excluded]

Paul worried that Deaf people would be excluded from teaching their language because of the barriers which exist to accessing education.

Finally, Richard expressed concern for the lack of available teachers for the Deaf in Northern Ireland.

Richard: [There is a] good number of teachers for the Deaf here who originally are from Northern Ireland, but they have no choice but to go over to England to be able to continue their career as teachers, and that’s because the system doesn’t work here for them.

This is particularly a concern when considering regional variation in sign languages—those competent in Northern Irish variant sign language are discouraged from remaining in Northern Ireland.

### Acquisition of other language and literacy in Deaf adults

Participants in the UK expressed concern about access English when Deaf children leave school and subsequent insufficient education in Deaf adults.

Limited proficiency in English for Deaf children in the UK was an issue mentioned by Oliver and Alan.

Oliver: So you've got a sixteen-year-old hearing child left school, academically or educationally trained up at sixteen, whereas a Deaf child at sixteen has a reading age of eight*.*

Alan: So the average reading age of a Deaf child leaving education is eight. And that's been the same for many years now. So why haven't we addressed that and done something about it? So the BSL act needs to have education within it*.*

Vivienne, who worked at an organization providing services for Deaf people in the UK, attested that many Deaf adults possessed a very rudimentary command of English:

Vivienne: A lot of clients within my workplace, when they come to us their language, it's just sort of entry level three, which is sort of seven- to eight-year-old language skills. And they’re maybe in their early forties, maybe late forties*.*

Vivienne was making the point that this lack of English could hinder the prospects of Deaf adults.

Proficiency in English was also mentioned in terms of access to other areas of education by Richard:

Richard: Because you might be aware that Deaf children and young people are less likely to have five GCSEs or more, compared to their hearing peers, and again that’s down to lack of access in languages*.*

As noted at the outset, language deprivation and sensorial asymmetry are problems in the Deaf community (De Meulder et al., 2019; Gulati, 2019). As discussed in the previous two sections, quality of education and a lack of appropriate teachers may also impact a student’s ability to learn and thereby acquire other languages. This may an impact on their capabilities in other areas. For example, if a school geography or history textbook is written in English, a Deaf child with limited English will struggle to access that material because of their lack of English, thereby impacting their ability to learn across the spectrum of education. This can then contribute to the lower attainment levels that can be seen in Deaf schools but which are not typically caused by being Deaf (Dammeyer & Marschark, 2016).

### Illusion of choice

Many participants alluded to the availability and quality of Deaf schools in contrast to mainstream schools. Often the Deaf schools were located far away from participants or were lacking in quality education as described above. Whereas mainstream education options (i.e., being educated in a hearing school, with assistive technology or an interpreter). This can mean that parents are unable to make a meaningful choice between mainstream education and a Deaf school. Additionally in the UK, if parents wish to send their children to bilingual schools, the pool of options is limited (see Table 1) and centralized to England.

Paul was disappointed that the BSL Act would not have any impact on this issue for families with Deaf children.

Paul: You know, so, “Well, you have the choice where you can go to,” and the choice is… you have a five year old child and get them to boarding school and you see them once at half term, or you put them in a mainstream school where they don’t have access. That’s no choice! So again, the BSL Act changes none of that*.*

Note that all of the bilingual schools listed in Table 1 are located in England. Therefore, a Welsh, Scottish, or Northern Irish family would need to relocate or send their child to board at a school far away in order to access that type of education. This point was expressed by Richard:

Richard: …we know we don’t have a school here in Northern Ireland that specialises in sign language teaching, we only have Jordanstown School for the Deaf and the Blind, but that school for me at the moment is a school for Deaf children with additional needs, OK? So if there are other children who are Deaf only, they find themselves in a position where they maybe have to go into mainstream school*.*

For parents of Deaf children in Northern Ireland then, the one school is not necessarily appropriate for all families. Richard went on to note that if parents did wish to send their children to Deaf schools in England, they would need to pay for this themselves:

Richard: But now obviously with the local authorities here in Northern Ireland, they're not able to pay fees, so they decided to scrap the idea of sending a child off over to England for better schooling. Now as a result, we find that most Deaf children in Northern Ireland, they stay here in Northern Ireland…. So that’s been quite difficult for parents, when they’ve ended up with no choice but to put their child into a local school, hoping that they have the right support available, to make sure that the child can progress in school*.*

Children in Northern Ireland then can experience an unequal provision of services depending on their geographical location, wherein some schools may provide excellent services, but others may be lacking. This sentiment was shared by Mikko, in reference to schools in Finland:

Mikko: And I struggle to understand why Deaf parents [in Finland] don’t want to send their kids to the Deaf school and instead get their children to a hearing school. Because you cannot really—you can’t guarantee that their quality of interpretation is always great in a hearing school either*.*

Aino referenced her own experiences as the parent of a Deaf child and deciding against mainstream school.

Aino: And we didn’t have that many good options, so basically our family needed to decide what is important for our kid and we decided that we did not want our child to go to school at that young age just to use an interpreter. They are fluent with Finnish Sign Language and use an interpreter as well, they are really skilled with that, but still we didn’t want them to go to mainstream school and have just one signing person, one signing adult in their life who was in that school*.*

Recall that there are now no Deaf schools in Finland. Finland is a large country. The distance from the northern city of Rovaniemi to Helsinki is over 800 km. The choice of a family with a Deaf child in Rovaniemi is to relocate to Helsinki or send their child to a local school and hope for appropriate supports. Additionally, Aino points to the potential experiences for a Deaf child who uses an interpreter in an otherwise hearing school—they are the only Deaf child, or one of only a few. They may struggle to have natural interactions with their peers on the playground or in non-classroom settings (Selin-Grönlund, 2021). The constant need for an interpreter has the potential to impact their social interactions with peers. For a Deaf student in a bilingual school, however, they are free to communicate directly in sign language with their peers and build relationships as one might expect of children.

Again, we can see how the illusion of choice issue is connected to all of those issues which have arisen to this point; quality of education, systemic problems such as Deaf schools being conflated with disabled schools. If a parent is concerned about the quality of education at a particular school, they may worry about their child’s acquisition of other language and decide to send their child elsewhere. Indeed, the illusion of choice is also connected to the following issue about the dwindling number of Deaf schools.

### Dwindling number of Deaf schools

Participants in the UK and Finland referenced the dwindling number of Deaf schools. In Table 1, the breakdown of Deaf schools across the UK, including their educational philosophy is shown. In Finland, many Deaf schools have closed in recent years, leaving few options for Deaf children.

Tiina and Aino referenced the lack of Deaf schools across Finland:

Tiina: … across Finland, before we had many Deaf schools, whereas now we have no school. I think we don't have any schools that are call themselves a Deaf school. It's all become mainstream. And in Helsinki, [there is] one school with, I think there's five or six classes and they use sign language*.*

Aino: In principle we don’t have Deaf schools in Finland per se, we have classes that have pupils who have special needs, and their teacher who teaches those classes, they have to have education in special needs education*.*

Tiina also noted the lack of daycares:

Tiina: And the daycare, there's not one single daycare for Deaf children [in Finland]. There's not a government provision for that*.*

It should be noted that in the time since this interview took place, a bilingual daycare has opened in Helsinki which sets aside places for deaf children who sign at home. The daycare is open to Deaf and hearing students and uses Finnish and SVK (Helsinki Opens Bilingual Daycare Where Children Use Sign Language and Finnish, 2025). This is a positive, but at present, there is only one such daycare. The impact of lack of sign language access from early education has been mentioned to above, and can go on to impact the quality of education in Deaf schools where students can come to school with extremely varied language proficiency (Shaver et al., 2014).

In the UK, one participant noted the lack of Deaf schools in Scotland:

Peter: There are no Deaf schools. There are no communication supports, because Deaf children are being put into mainstream classes, so they might have no interpreters in the classes. I'm not saying there's no Deaf teachers, there are lack of BSL fluent teachers, there's a real lack of that*.*

As noted above, there are no Deaf schools in Wales. The Deaf school in Northern Ireland is specialized to DeafBlind and blind students. The schools in Scotland use a total communication method of teaching, rather than bilingual education in BSL and English.

Aino suggested that the increase in cochlear implantation across Europe as one cause for the decline in Deaf schools and the popularity of mainstream education While it is not the focus of this paper to debate the ethics or success of cochlear implants, it is known that sign language acquisition in no way hampers the ability to acquire other languages. In fact, it has been shown that sign language exposure can increase cognitive and linguistic abilities in implanted Deaf children (Delcenserie et al., 2024):

Aino: …in Europe, there has been… you could say, a neo-oralism or something of the sort…In Nordic countries, I think it’s seventy to – from the seventies to the two thousands, most of the Deaf people went to Deaf schools. Of course, the level of sign language varied in the teachers and children as well, but still they got the education in sign language. I have a friend in [a different European country], and I met them a while ago, and… they told me that they have met quite many people in their life who have gone through education without sign language and have not been able to sign until they have been grown up. But in Finland that hasn’t been the case, so far. There are quite – just a few people who have gone through education, through that. We have had, traditionally, just a few late signers. But that is about the change, I think, because of the cochlear implant situation and the education system*.*

This “neo-oralism” meant that children were being denied access to sign language because of assistive technology. While “neo-oralism” is a term typically used to describe a system of Deaf education developed in the Soviet Union which involved fingerspelling and oral communication (Moores, 2010), Aino is referring to a different concept. Although oralism is no longer the educational philosophy, a shift away from Deaf schools and institutions has meant that Deaf people can no longer access signed language. This may be similar to the concept of “reinstitutionalizing” the Mathews describes, which says that although the physical institutions which once housed and educated Deaf people using oralism have gone away, the process of isolating Deaf children from each other in mainstream schools ill-equipped to handle the linguistic needs of Deaf children has effectively completed the same process as oralism (Mathews, 2011, 2017).

Again, the interconnectedness of the concerns of participants is evident here. The dwindling number of schools is directly relevant to the illusion of choice discussed. The lack of availability of Deaf preschools means that many children who do not have a signed language at home will have little to no signed language when they begin school, which can impact their ability to acquire other language, and interfere with the quality of education as discussed. And there are systemic problems, such as the lack of appropriately trained teachers which may impact the ability of a Deaf school to remain open, thereby contributing to their decline. The problems with education as expressed by respondents are multifaceted but interconnected.

Moving forward, I will address RQ2 and present data which shows what changes or improvements to the provision of education for Deaf people in Finland, Ireland, and the UK are suggested by Deaf people in those countries.

### Meaningful access to sign language education

Each participant was asked if they might wish to change law or policy for the benefit of the Deaf community in their state, and if so, what would they change. Participants suggested how education systems might be improved. Chiefly, they suggested a need for meaningful access to sign language education for the Deaf community. Better access to sign language education was suggested by participants in all three jurisdictions.

While Meghan was based in the UK, here comments referred to all Deaf people:

Meghan: Deaf people should have the right to bilingual education

According to Meghan, Deaf people deserve the right access both English and signed language. This is a call for bilingual education, as opposed to education using total communication, oralism, mainstreaming, or other forms of education.

Many participants stated they wanted the Deaf children to have the express opportunity to access sign language:

Alan: Deaf children need the opportunity to learn via their own language. Their preferred language

Paul: I suppose if I always had a say, the one thing I would talk about would be, you’d have to have genuine options for Deaf kids to have full education through BSL…If that was possible that children had genuine, equal and full access to education, you know, from year dot right through to as you exited, I think that would change everything

Dara: I do think something clear should be put in there [into ISL Act] about Deaf children's access, nothing about assessment or anything like that, nothing that talks about hearing loss, just the rights of Deaf children to access sign language.

Tiina: At birth, maybe the parents don't sign, so it should be that teachers are provided. So you can learn then language from your parents. And then at daycare you should have exposure to Deaf children and Deaf adults. There should be sign language in daycare. And then the same when you go to school, there should be good quality education in schools in sign language.

Charles: I’d want a statutory register of BSL teachers. I’d want a nought to five early years intervention scheme widely known. We know that 90% of Deaf children are born to hearing parents…importantly what's missing right now is that Deaf intervention sign language intervention and to see bilingualism.

It is clear that a major concern for participants was access to meaningful, quality bilingual education. In fact, no participant in all of the interviews expressed any desire for any other type of education (total communication, mainstream, special education, etc.).^4^ Certainly there were criticisms of mainstreaming and total communication as methods, typically based on participants’ own lived experiences in those systems. When participants expressed a desire for improved education, they were exclusively referring to bilingual Deaf education. Tiina summarized the preferred experience for Deaf children:

Tiina: … Deaf children are different. Deaf children have different needs. They need other Deaf children who can sign. They need Deaf adults. They need Deaf staff to teach them. They need that a support from Deaf people who can sign so that their language can develop*.*

Deaf children, according to Tiina, need a Deaf community, a signing community, adult role models and peers who can communicate with them, in their natural and native languages.

From the data, we can conclude as a response to RQ2 that the participants expressed a desire for better, meaningful, and complete access to bilingual education for Deaf children. However, as the findings of RQ1 show, there are complex and multifaceted issues which pose barriers to acquiring such meaningful, complete access.

## Barriers remain

It is important to note, however, that access to education in signed languages alone is not sufficient to override inequalities experienced by Deaf people. As one participant succinctly put it:

Meghan: Even if I had sign language all of my life, I’d still be facing barriers.

There are still barriers in place, outside of the provision of education. But undoubtedly, access to appropriate and quality education is a start.

Tim: And I think also education, isn't just something that can be seen in silo. It's not just— it's looking at the holistic, looking at it from a holistic manner. Because you're looking at the child, the family environment, what language the family may use, external influences. So there's many people that have been talking about that and over Deaf children growing up and not having access to language. And then they do when they're in school but it's almost as if you're playing catch. So also what happens during the language development stages of a child if they don't have that exposure? So again, it's very complicated part of it's many moving parts that you've stated, but of a massive, massive picture*.*

There must also be an assurance of quality education, where there are mechanisms in place to assess that quality. Additionally, there needs to be more Deaf schools available so that families are not forced into a choice between mainstream education or sending their child to live hours away to access sign language education. The Deaf people in this study were clear in their preference for quality bilingual education, for the need for meaningful access to sign language for Deaf children and for that access to come even before a child goes to school. The problems are multifaceted and interconnected, and therefore, require multifaceted solutions. Improving the quality of education at Deaf schools will mean ensuring that there *are* Deaf schools to improve. It will mean ensuring that Deaf children have access to language *before* they attend school. It will mean that teachers are educated and trained appropriately to meet the needs of *Deaf* students. It will mean that teachers have signed languages and that Deaf people can themselves *become* teachers. None of the laws which recognize signed languages in Ireland, Finland, and the UK are meeting the educational needs of the respective Deaf Communities. Deaf Communities sought to be provided with education, but also quality education, meaningful, and accessible education. There must be a recognition of the barriers in place when Deaf communities try to access education, and real efforts to overcome those barriers.

## Discussion

This article proposed research questions, which asked

RQ1: How do Deaf people in Finland, Ireland, and the UK perceive the status of education for Deaf people?

RQ2: What changes or improvements to the provision of education for Deaf people in Finland, Ireland, and the UK are suggested by Deaf people in those countries?

In answering those questions, we can say that while bilingual education provided through the medium of sign language is provided in all states, the availability of these education institutions is limited and seems to be on the decline. While mainstream education or other education options are available, it is notable that in all three countries, bilingual education in sign language does not seem to be the dominant form of education for Deaf children.

In addressing RQ1, participants were concerned about the quality of education for Deaf children. Quality of education in Deaf schools is a multifaceted problem with many contributing factors as discussed by participants and shown in the corresponding literature (Dammeyer & Marschark, 2016; Knoors & Marschark, 2014; Mathews, 2011; Shaver et al., 2014). The quality of education is connected to the systemic problems that participants referenced. Deaf people themselves experience barriers in education, which can have a knock-on effect and prevent them from entering the workforce or becoming education providers in Deaf schools. Systemic barriers prevented Deaf people becoming teachers, favoring teachers with training in special education over Deaf education and minimizing emphasis on the need for fluency in a sign language among teachers. The quality of teaching itself then has an impact on the provision of quality education (Dammeyer & Marschark, 2016). These problems then evidently impact on children’s ability to acquire literacy, which can in turn limit their educational potential and future job prospects in a hearing-dominant world.

Finally, although there may be the perception that parents have a choice about which schools and which educational philosophies to choose for their Deaf children, systemic and geographic barriers mean that this choice is an illusion. Where it is known that the quality of education inside Deaf schools is poor, or the nearest Deaf school is hours away from the family home, there element of choice for parents is greatly reduced. The dwindling numbers of Deaf schools available and the societal push toward mainstream education as an appropriate solution for Deaf students also impact parental choice (Mathews, 2011).

In addressing RQ2, Deaf people showed a desired that the laws which are introduced to protect Deaf people and provide for sign language recognition provide for bilingual education, so that Deaf people may have lifelong, complete, and well-rounded access to sign language and education. There was no express desire for mainstream education, and in fact, there was an acknowledgement by some that mainstream education was undesirable, similar to what Mathews suggests in her work on reinstitutionalizing Deaf children through mainstream education (2017) or to what Selin-Grönlund has observed about Deaf children’s inclusion in mainstream education by their peers (2021). Without access to equitable, quality education, among peers and Deaf people continue to experience discrimination and marginalization. RQ2 asks what changes or improvements to the provision of education for Deaf people in Finland, Ireland, and the UK are suggested by Deaf people in those countries. In answering this questions, participants to this research wanted accessible, meaningful, and long-term bilingual education for their communities.

This article in no way aims to suggest, however, that the improvement of education systems for Deaf people alone will ensure that Deaf people no longer experience inequality or discrimination. Barriers remain and will remain, even if education systems are overhauled overnight. Improvements in access to education, quality of education, availability of education will take years to show concrete improvements. And in the interim, Deaf people still experience discrimination in workplaces, legal settings, healthcare and in their day-to-day lives beyond the education system.

## Limitations and further research

Certainly, this research may have benefited from a broader international study, with input from non-European, non-Western perspectives. Further research is necessary to investigate if the findings in this research are reflected on a larger international scale. Additionally, the language capabilities of the principle investigator on this project meant that collected data could not be analyzed in its source language and member checking was not possible. Only the interpreters booked for interviews were responsible for all of the translation and although the interpreters were all highly trained and qualified, there is potential for bias or inaccuracies therein. It may also have been beneficial to collect more data biographical on the individual interviewees, and a different methodology might have allowed for this type of interviewing. While coding was done by an experienced researcher, a larger research team could have allowed for a second person to establish intercoder reliability. Finally, although there were many participants to the research, it would also have been of benefit to seek out more people, but for time, cost, and logistical constraints, this was not possible in this particular research project.

## Conclusion

As noted at the outset, access to education of any kind is a rarity for Deaf people globally. That is not to say, however, that Deaf people who are in receipt of education must be happy with their lot, that they must accept the education given to them, even when that education is below the standard given to hearing children (Human Rights of the Deaf - WFD, 2024). Deaf people deserve adequate, appropriate, meaningful access to education in their own language as a right. It is wholly possible to guarantee this right in state legislation or even at constitutional levels. But doing so requires meaningful engagement with and listening to Deaf communities, their needs and wants and long term, systemic changes to effectively implement provisions to achieve those needs and wants (Ladd, 2003; Mathews, 2017). The Deaf people in this study were clear in their preference for bilingual education, for the need for meaningful access to sign language for Deaf children and for that access to come even before a child goes to school. It was clear that the laws in Ireland, Finland, and the UK recognizing sign language are not meeting the educational needs of the respective Deaf Communities. Deaf Communities must be provided with education, but also quality education. There must be a recognition of the barriers in place when Deaf communities try to access education, and real efforts to overcome those barriers. There needs to be improvements in the methods of teaching, so that children and Deaf adults can access other language at an age-appropriate reading level. There must be more Deaf schools, where bilingual education is meaningfully provided, so that parents can make real choices about what is best for their Deaf children where teachers are equipped and prepared to provide such education (Selin-Grönlund, 2021). Until Deaf people are listened to, and their needs and wants are understood and respected, laws will continue to fall short of actually providing meaningful, impactful, and desirable changes for the Deaf community.

## Footnotes

1 For a more detailed understanding of the history of the two SLs in Finland, see Takkinen et al. (2015) and Scheinin et al. (2004).

2 Not to be confused with Deaf phenomenology, which is as separate philosophical framework.

3 The General Certificate of Secondary Education, abbreviated to GCSE is a series of academic examinations across a range of subjects taken by second level students across Northern Ireland, England and Wales.

4 It should be stressed that participants were not asked outright whether they preferred bilingual education, or for their preference of education at all.
